# Using human *in vitro* transcriptome analysis to build trustworthy machine learning models for prediction of animal drug toxicity

**DOI:** 10.1038/s41598-020-66481-0

**Published:** 2020-06-12

**Authors:** Laura-Jayne Gardiner, Anna Paola Carrieri, Jenny Wilshaw, Stephen Checkley, Edward O. Pyzer-Knapp, Ritesh Krishna

**Affiliations:** 1 IBM Research UK, Sci-Tech Daresbury, Warrington, UK; 2 STFC Daresbury Lab., Warrington, UK

**Keywords:** Drug discovery, Machine learning

## Abstract

During the development of new drugs or compounds there is a requirement for preclinical trials, commonly involving animal tests, to ascertain the safety of the compound prior to human trials. Machine learning techniques could provide an *in-silico* alternative to animal models for assessing drug toxicity, thus reducing expensive and invasive animal testing during clinical trials, for drugs that are most likely to fail safety tests. Here we present a machine learning model to predict kidney dysfunction, as a proxy for drug induced renal toxicity, in rats. To achieve this, we use inexpensive transcriptomic profiles derived from human cell lines after chemical compound treatment to train our models combined with compound chemical structure information. Genomics data due to its sparse, high-dimensional and noisy nature presents significant challenges in building trustworthy and transparent machine learning models. Here we address these issues by judiciously building feature sets from heterogenous sources and coupling them with measures of model uncertainty achieved through Gaussian Process based Bayesian models. We combine the use of insight into the feature-wise contributions to our predictions with the use of predictive uncertainties recovered from the Gaussian Process to improve the transparency and trustworthiness of the model.

## Introduction

In order for a pharmaceutical drug candidate to progress to the human trial phase, regulations require preclinical trials to ascertain the safety of the compound. These preclinical trials commonly involve testing on animals such as dogs, mice, rats and rabbits. However, although toxicity in animals can translate to human toxicity, this translation shows considerable variability^1,2^. It is important to understand the degree to which biological perturbations that are observed in animals can be translated to humans^3^. This is balanced against the desire to reduce the overall requirement for testing compounds on animals wherever possible due to rising expense and ethical implications.

Machine learning (ML) offers a path to derive insight on this animal to human translation for drug toxicity^4^. Transcriptomic data can be combined with machine learning methods to predict adverse effects after compound exposure and much research to date has focused on predicting a compounds toxicological class or endpoint^5–8^. However, this is commonly assessed in human or rat models translating between *in vivo* and *in vitro* experimental measurements. Here we translate between species and experimental differences, we use ML to ascertain whether *in vitro* human transcriptome data can be used for predicting compound toxicity *in vivo* in animals such as rats – a model drug testing species. In order to enable this methods such as RNA-seq can be used to profile the transcriptome in full to understand compound-induced toxicity. Studies have also demonstrated the integration of multi-omics data for toxicology studies^9^. However, such approaches are expensive when there are a large number of compounds to screen, as is typical in the early stages of drug development, and the resultant data requires a considerable amount of time, computation and expertise to process^10^. As such, there has been a large amount of effort invested in cost effective experimental methods that can generate datasets from which the likely effect that a compound will have after its administration, particularly with respect to toxicity or adverse events, can be predicted. One such approach is L1000, a high-dimensional gene expression profiling method, which is fast emerging as a cheap alternative to produce large amounts of experimental observations that are suitable for machine learning driven drug discovery^11^.

In this study, we use human cell-line derived L1000 gene expression or transcriptomic profiles that have been generated for a variety of chemical compounds^12^. We apply machine learning to L1000 profiles generated after chemical compound treatment, to predict specific rat phenotypes associated with kidney dysfunction that are induced after treatment with the same compound (Supplementary Fig. S1). Impairment of renal function or drug-induced nephrotoxicity is reported as a common adverse drug effect that is linked with systemic toxicity of a drug^13^. Therefore, using regression, we predict a continuous variable, the amount of blood urea nitrogen (BUN) in rats, as a proxy for drug induced renal toxicity, since elevated levels (28–136 mg/dl) are routinely used as an indicator that the kidneys may be damaged or dysfunctional^14,15^.

We predict BUN levels using either gene expression data, compound chemical structure information or both datasets as features. Combining gene expression data with chemical structure information allows us to substantially improve predictions, in line with previous work^12^. As the combined feature space exceeds the number of observations, as can be typical for genomics datasets, we use dimensionality reduction to tackle problems associated with a sparse and high dimensionality dataset. We also highlight the benefit of using Gaussian processes to understand the context of a prediction in terms of its transparency and trustworthiness, which is of high importance in healthcare. Here, we integrate heterogeneous experimental datasets and, using ML we predict rat blood tests (*in vivo*) as a proxy for renal drug-induced toxicity using *in vitro* inexpensive human cell line tests. Our combination of *in vitro*-to-*in vivo* translation with human-to-mouse inter-species translation for toxicity prediction assists our ultimate aim to identify translatability between species and to reduce animal testing in drug development by identifying likely toxic compounds earlier in the development process or before animal testing. This supports the pharmaceutical industry’s commitment to the 3 R’s (Replacement, Reduction, Refinement) in drug development^16^.

## Results and Discussion

### Dataset enrichment through augmentation of L1000 features with chemical structure information

We propose that a richer feature set, when coupled with an appropriate model, should improve the predictive power of the approach. Biomedical data, of the type studied here, often suffers from a lack of expressiveness, which has hindered the uptake of machine learning approaches. As such, in this study we augmented the L1000 features with chemical structure information to provide a richer feature set, which we hoped would circumvent this challenge and improve predictive power. To test this, we applied Gaussian Process (GP) regression^17,18^ to predict rat BUN level using as training data; firstly, only the L1000 gene expression profiles (964 features), secondly, using only chemical structure information (166 features) and finally, combining both resources (1,130 features). For all three analyses we used a kernel which was the sum of a simple RBF kernel, and a WhiteKernel. Hyperparameters were optimized using gradient descent on the marginal log-likelihood of the model. Due to the large dimensionality of the data, a single length scale was used over all features.

**Figure 1:**
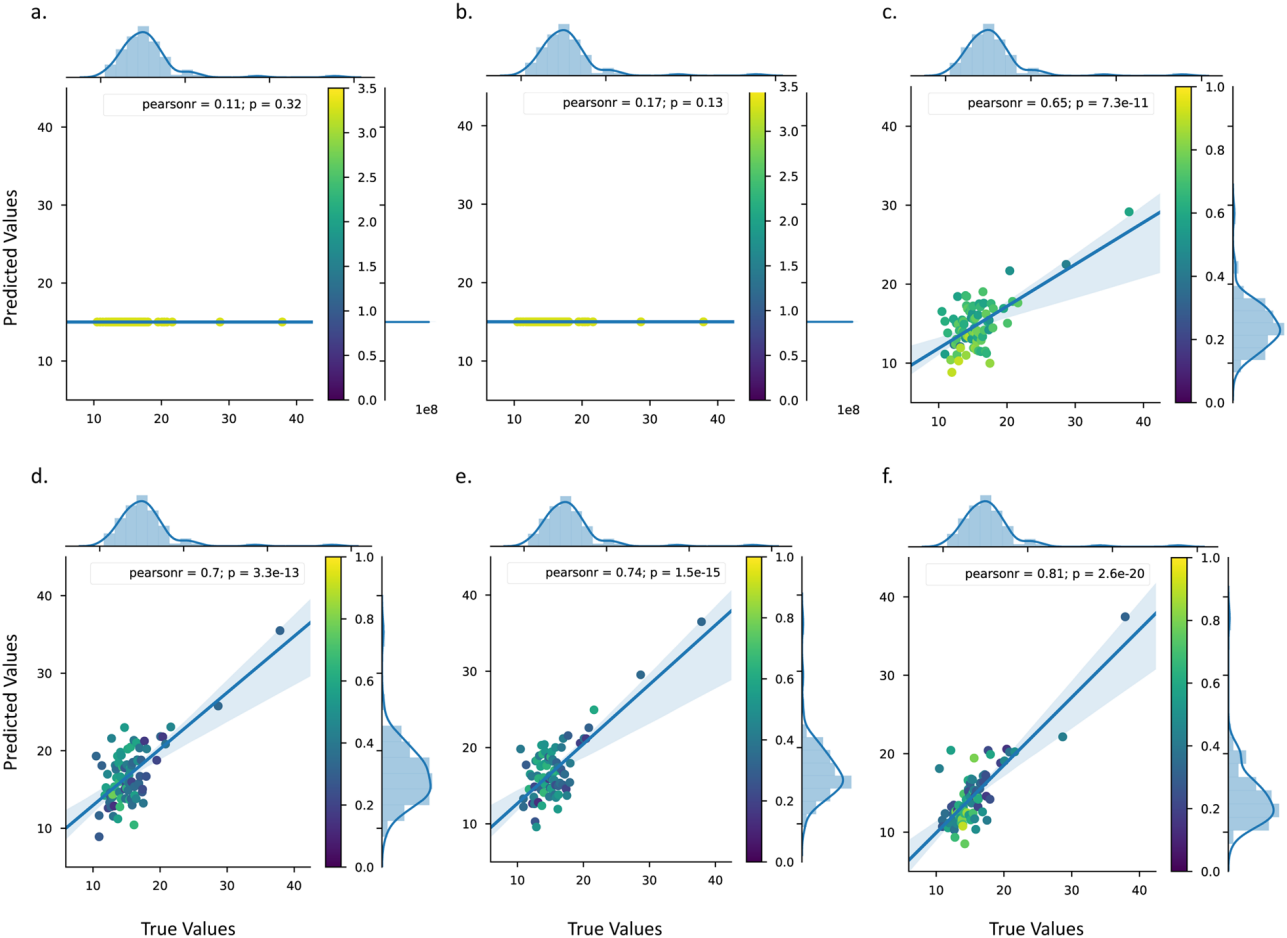
True versus predicted test values from BUN level prediction. Scatter plots showing the true (x-axis) versus predicted (y-axis) test values using: **(a)** gene expression data as training data, **(b)** chemical structure information as training data and **(c)** both gene expression and chemical structure as training data. Scatter plots showing the true (x-axis) versus predicted (y-axis) test values using gene expression and chemical structure as training data after: **(d)** PCA using 57 components, **(e)** tSVD using 57 components and **(d)** tSVD h. All scatterplots show marginal histograms with regression and kernel density fits. The 95% confidence interval for the regression estimate is drawn using translucent bands around the regression line. Datapoint colour is according to the standard deviation of the predictive distribution per point; scales for colour vary between plots. Figure **(f)** shows our best model.

Figure 1(a–c) and Table 1 show that the combination of gene expression and chemical structure information decreases test set RMSE scores to 2.961 from the average 3.708 seen separately. There is also a marked increase in the test set r2 scores after combination of the features from on average 0.021 to 0.418. Figure 1 highlights the true versus predicted test values where, correlation coefficients increase from 0.11 and 0.17 for gene expression and chemical structure separately to 0.65 after combination. Furthermore, Fig. 1 shows that prediction confidence increases after feature combination with the average uncertainty level (the standard deviation of the predictive distribution for each test datapoint) decreasing from 3.283 for gene expression and chemical structure separately to 0.683 for the combined features.

**Table 1:** ML analyses to predict BUN levels in rats.

Training dataset	L1000 gene expression	Chemical structure	L1000 gene expression plus chemical structure	L1000 gene expression plus chemical structure	L1000 gene expression plus chemical structure	L1000 gene expression plus chemical structure	L1000 gene expression plus chemical structure
Regressor	Gaussian Process	Gaussian Process	Gaussian Process	Gaussian Process + PCA 57	Gaussian Process + tsvd 57	Gaussian Process + tsvd hierarchical	Light GBM
Test set MAE score using best parameters	2.156	2.156	2.365	2.700	2.452	**1.710**	1.544
Test set RMSE score using best parameters	3.708 **3.708	3.708 **3.708	2.961 **2.240	3.359 **2.157	3.176 **2.047	**2.419** **1.528	2.146
Test set r2 score using best parameters	0.013	0.028	0.418	0.464	0.520	**0.661**	0.656
Average SD of the predictive distributions (ASD)	3.283	3.283	0.683	0.427	0.425	**0.470**	—

Showing the results from the analysis to compare the effect of combining chemical and gene expression data, the effect of using dimensionality reduction and finally comparing the best alternative classifier from the 8 tested, defined as producing the highest test prediction accuracy (lowest MAE balanced with highest r2 score). The best model overall is highlighted in bold. **Weighted RMSE.

The combination of gene expression and chemical structure information increases our predictive capability for BUN level. This analysis also highlights the benefit of using a GP with the ability to directly capture the uncertainty of the predictions since there is a dramatic decrease (4.8-fold) in the uncertainty level after feature combination while the difference in RSME scores was not so marked (1.3-fold) and could mask a poorly performing model.

### Dealing with high dimensional data where d ≫ N

One of the main challenges presented by this dataset, and many others in the field of genomics, was a far greater number of features than datapoints (1,130 features vs 429 datapoints). As such, traditional methods would be susceptible to overfitting since any parametric model where d > N will, without proper regularization, produce a perfect fit to the training data, but fail to generalize. We addressed this using two approaches, firstly, using GP and secondly by using dimensionality reduction techniques. Because GP uses a covariance matrix, rather than the input features themselves, in its prediction, the predictive power scales with the number of data points rather than the dimensionality. Here, after investigation, we found a kernel comprising of a sum of the common Radial Basis Function (RBF) kernel, with automatic relevance determination (ARD) to best model the data. This is expressed as:

$$k({x}_{i},\,{x}_{j}|\theta )={{\sigma }_{f}}^{2}exp\left[-\frac{1}{2}\mathop{\sum }\limits_{m=1}^{d}\frac{{({x}_{im}-{x}_{jm})}^{2}}{{l}_{m}^{2}}\right]$$

where ${({x}_{im}-{x}_{jm})}^{2}$ represents the squared distance between two feature vectors, ${l}_{m}^{2}$ the characteristic length scale of each feature, ${\sigma }_{f}$ is the signal standard deviation. It is clear from this, however, that problems with large, sparse, descriptors can creep into the kernel of a GP through the distance term. This is because many standard distance measures will not take the nature of the vector into account, and thus simply the number of zeroes in such a vector will ensure a small distance. There are two potential avenues to tackle this problem – either by using a distance measure which understands the sparse nature of the input features, or through a dimensionality reduction approach.

Dimensionality reduction has been shown to have a significant effect in building powerful machine learning models from genomics data such as gene expression datasets that have similar dimensionality characteristics to our dataset^19,20^. Thus, we reduced the dimensionality of our problem using a similar approach to that demonstrated previously^21^ utilizing a truncated singular value decomposition (t-SVD)^22^. The use of t-SVD is necessitated by the fact that the feature matrix is not square (i.e. N! = D), and so we cannot simply use eigenvalues and eigenvectors to decompose the matrix. Since we can write our SVD – decomposition of a matrix A as

$$A=U\Sigma {V}^{\ast }$$

and thus,

$$A=\mathop{\sum }\limits_{i=1}^{p}{\sigma }_{i}{u}_{i}{\upsilon }_{i}^{\ast }$$

where ${\sigma }_{i}$ are the singular values, and ${u}_{i}{\upsilon }_{i}^{\ast }$ are the i-th column of U and V, respectively we can imagine a k-truncated approximation as

$${A}_{k}=\mathop{\sum }\limits_{i=1}^{k}{\sigma }_{i}{u}_{i}{\upsilon }_{i}^{\ast }$$

which, under the Eckart-Young theorem^23^ represents an optimal solution at all values of k, where the singular value of k + 1 represents the 2-norm error of the compression.

Since our feature space is represented by two different sets of features (the L1000 profiles, and the chemical descriptors), we chose to implement a hierarchical approach to t-SVD (t-SVDh), where the two parts of the feature space were considered separately, and then recombined. Preserving 95% of feature information per sub-domain resulted in a 57-dimensional vector, with the first 17 dimensions representing chemical features (reduced from 166 dimensions) and the final 40 dimensions representing the L1000 profiles (reduced from 964 dimensions). We tested this hierarchical tSVDh approach against a straight dimensionality reduction to 57 dimensions using both t-SVD and the commonly used PCA method (Fig. 1(d–f), Table 1). tSVDh gave a very strong correlation coefficient of 0.81 and r2 of 0.661 exceeding any other observed with this dataset and alongside the lowest weighted RMSE of 1.528. Importantly, all of these methods outperformed a GP with no dimensionality reduction applied to it.

### Capturing Model Uncertainty to Improve Trust and Transparency

Within sensitive domain areas, which are epitomised by medical and pharmaceutical applications, we believe that one of the major barriers to the uptake of ML, and more generally data-driven technologies, is a perceived lack of trust – sometimes referred to as the ‘black box problem’. We propose that a degradation of trust in a model, or an approach, can come from two major sources: poor predictions on out of set problems and lack of transparency and interpretability. To address poor out of set modelling, we build a rich description of the model uncertainties and return both a prediction and an associated confidence measurement. Thus, an out of set prediction will come with an inbuilt ‘warning’ about possible performance issues and can be treated accordingly.

It is important that predictive uncertainties are sufficiently descriptive, otherwise they add very little information about the quality of the model. In an ideal world, we want points which are poorly predicted due to out of set character to also have high uncertainty associated with the prediction. As we have demonstrated previously, the dimensionality and nature of the features can strongly impact the quality of the model; and the same is true with the quality of the uncertainties. Our ‘full’ feature set (i.e. features to which no dimensionality reduction had been applied) did not display our desired behaviour, with large uncertainties throughout the test set. However, when we use our hierarchical dimensionality reduction, we observed much lower levels of uncertainty.

**Figure 2:**
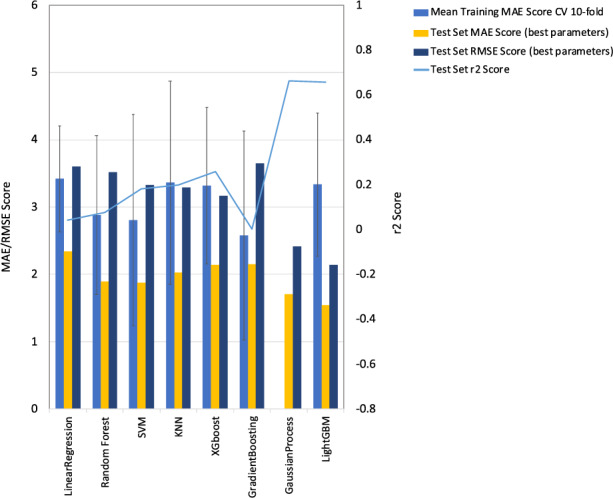
Results from BUN level prediction ML comparative regression analysis. Bar charts showing the RMSE and MAE scores for the test datasets (using best parameters) and the mean MAE training scores after 10-fold cross validation with the standard deviation shown as error bars. Left y-axis is used for MAE/RMSE scores. Line plots show the R^2^ score for test datasets (using best parameters) with the right y-axis used for R^2^ scores. For Linear Regression, SVM, KNN, XGBoost and GP tSVDh dimensionality reduction was used whereas for other approaches it was not since resulting models were more predictive without it.

To demonstrate the importance of predictive uncertainties, we also fitted commonly used deterministic methods to the data and compared the results to our best GP model with and without dimensionality reduction for comparison (Supplementary Tables S1 and S2; see Methods). Here, for several of the methods we found the resulting models to be more predictive using the reduced feature set for predictions (Linear Regression, SVM, KNN and XGBoost) while others did not benefit from dimensionality reduction (Random Forest, Gradient Boosting and Light GBM). There is overlap between the training set MAEs (Mean Absolute error) ±1 SD (Standard Deviation) after cross validation to the test set MAEs (Supplementary Table S1), for all models except for LightGBM, however, the LightGBM difference between train and test MAE represents only 5% of our full target range (7.75–39.60 mg/dl) suggesting that overfitting of the models is not an issue. Using our best models for each method considering r2 score, MAE and RSME (Root Mean Squared Error), our GP model performs comparably to other algorithms with its closest rival model generated with LightGBM (Fig. 2, Table 1). However, the inclusion of uncertainty measurements with GP also allows us to calculate a weighted RSME. The low uncertainty rate from our best GP model (0.470) results in the lowest observed RMSE (1.528) across all models after weighting and the highest r2 score of 0.661 (Table 1, Fig. 1f). Furthermore, the inclusion of uncertainty measurements with GP gives additional benefit. We highlight a specific sample with a BUN level of 37.89 mg/dl consistent with potentially severe kidney dysfunction being ~10 mg/dl over the threshold for elevated BUN. For this sample, the LightGBM model predicts a BUN level of 34.61 mg/dl decreasing it by 3.3 mg/dl. In contrast, the GP predicts 37.23 mg/dl representing a 5-fold prediction improvement. Additionally, the GP’s low uncertainty estimate of 0.661 confirms the close proximity of the prediction to the true value. Here, the absence of this extra information for the LightGBM model, could result in a case of severe kidney dysfunction that may need immediate intervention, being mis-diagnosed for a more moderate condition.

**Figure 3:**
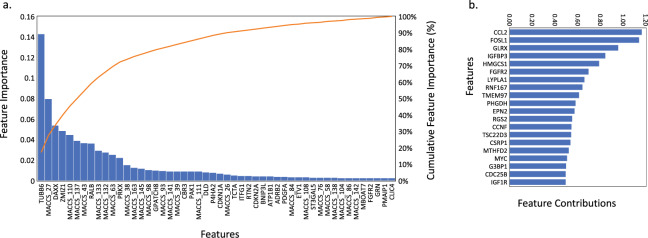
Investigation into feature importance for ML model. Showing the results from **(a)** the ExtraTreesRegressor approach, trained on L1000 gene expression data and chemical structure information. Showing feature importance as a bar chart for the 964 landmark genes and 166-bit MACCS chemical fingerprints, alongside a cumulative line plot of feature importance and **(b)** the highest feature contributions (>0.5) for the gene expression-based dimension with the shortest length scale in our best GP model.

Trust in a model, can also be improved by generating insight into the contributing factors to its predictions. To gain such insight into the genes whose expression profiles are most influential in our predictions, we investigated feature importance using two methods. Firstly, we used the ExtraTreesRegressor approach, trained on L1000 gene expression data and chemical structure information, therefore this does not consider the GP model. From this approach we observed that many features (632) contribute to the model at a significant level (>4E-09) (Fig. 3a). Overall 54.0% of cumulative feature importance is contributed by gene expression information and 46.0% from chemical structure information highlighting the benefit of combining the two information sources. Of the top 10 most important features, 4 are L1000 genes with little or broad relevance: TUBB6 (tubulin beta 6 class V) contributing 14.3%, DAXX (Death Domain Associated Protein) that regulates a wide range of cellular signaling pathways for both cell survival and apoptosis contributing 5.4%, ZMIZ1 (Zinc Finger MIZ-Type Containing 1) that targets the urea transporter SLC14A1 contributing 4.9% and RALB (RAS Like Proto-Oncogene B) involved in cell division and transport contributing 3.7%.

Secondly, we use the length scales of our GP that are fitted per feature to give insight into the impact specific features have on our model – the shorter the length scale, the higher the impact. Since we built our features using t-SVD, it is necessary to use an inverse projection to relate the reduced feature space to the original, physically interpretable, features. We achieve this by building a dummy feature vector in which only the features we want to examine are switched on, and then use the inverse transform of the 57 dimensional t-SVD matrix to regenerate a dummy feature in the full space, which we can then examine to determine the contributions. We followed this methodology for the dimension with the shortest length scale in our best GP model based on gene expression and focused on the genes that represent this reduced feature space. The top three genes that represent this reduced feature space have relevant roles including (Fig. 3b): CCL2 that is linked to renal damage^24^, FOSL1 that regulates cell proliferation and survival and GLRX that is known to be expressed in the kidney and to contribute to the antioxidant defense system. This analysis differs from the ExtraTreesRegressor approach since it considers the GP model that we have built and, as a result, the highlighted most important gene is more closely associated to the biological purpose of the model i.e. to predict renal dysfunction or toxicity.

## Conclusions

ML has an important role to play in toxicity prediction in the context of reducing animal testing. ML has its own challenges, arising from the data (sparsity, low data volume, lack of structure) as well as from the user (requirements for transparency, reliability and trust). Our approach combines computational techniques to predict BUN levels in rats from transcriptomics profiles derived from human cell lines. While doing so, we satisfy both the data and user centric challenges that hinder the adaptation of ML for problems like these. In our study, hierarchical t-SVD when used with GP displays superior predictive power and reduced uncertainty. The predictive uncertainties recovered from the GP improve the transparency and trustworthiness of the model, allowing the end users to understand when it can and cannot be used. Finally, we further improve transparency by providing insight into the feature-wise contributions to the prediction – a starting point for explainable AI for health and pharmaceutical related applications.

## Methods

### Development of ML feature sets

We trained ML models to predict compound induced rat BUN levels from compound chemical structure and L1000 gene expression profiles generated after application of the same compound to human cell lines. Our training dataset, L1000 gene expression profiles and chemical structures, was collated and processed by^12^ and encompassed 964 genes with L1000 gene expression information plus 166 features that encoded the chemical fingerprint for the chemical compound that was used to generate the gene expression profile. Our L1000 and chemical structure training dataset includes 31,029 perturbations (different compounds, cell lines and treatments) with information for 964 “Landmark” genes following small molecule treatment.

L1000 gene expression information is available as −1, 0 and 1 values to represent down-regulation, no change and up-regulation of a gene’s expression level^12^. We used a single dose and treatment of each compound tested to avoid redundancy between datapoints for the chemical structure feature set and also the L1000 dataset since multiple doses were found to yield similar profiles after conversion to −1, 0 and 1 values (90.0% of measurements show a standard deviation of 0 between doses and overall an average standard deviation between doses of 0.081). Therefore, for this analysis we used the highest dose and longest length treatment of each compound to maximize the observable compound effect in the human cell line L1000 signature. As such all 8 of the human cell lines (A375, A549, HA1E, HCC515, HEPG2, HT29, MCF7, and PC3) are represented in the final test set. Further work could attempt to more closely match the compound treatment dosage between the human cell line treatment and the rat treatment although, the different natures of these experiments make matching doses challenging. Chemical structure information for the small molecule compounds was in the form of a 166-bit MACCS chemical fingerprint matrix that was computed using Open Babel^25^.

### ML target dataset

Our target to predict, was rat BUN level (mg/dl) after compound administration that was available from DrugMatrix (https://ntp.niehs.nih.gov/results/drugmatrix/index.html). The datasets used here were generated after drug compounds were administered daily to rats at concentrations ranging from 0.0375–6000 mg/kg across timescales of 0.25–7 days dependent on compound composition. These targets are continuous variables ranging from 7.75–39.60 mg/dl and encompass a wide range from low or safe levels of BUN to high levels that significantly exceed known health indicator limits. We matched the compound induced L1000 gene expression signatures with rat blood test results where the same compound had been used. This allowed us to match L1000 profiles with 429 rat BUN level measurements and therefore 429 different chemical compounds have been included in the training set.

### ML application

We used Scikit Learn (v3.7) for the ML analysis^17^. 80% of the data was used for training and the remaining 20% of divergent compounds was held out for testing. 10-fold cross validation was performed on the training data. We trialed both ShuffleSplit and K-fold methodologies for cross validation (n_splits = 5 and shuffle = True) where we observed little difference between the two methods and no difference in our overall conclusions and as such here we report only the results using ShuffleSplit. The methods’ hyperparameters were optimized using a grid search to test a range of parameters for 8 regressors; Linear regression, Random Forest, Support Vector Regressor, XGBoost, Gradient Boosting, GP, KNN and LightGBM (Supplementary Table S3).

## Supplementary information

Supplemental information.

## Data Availability

The processed L1000 and chemical structure information datasets analysed during this study are available from https://maayanlab.net/SEP-L1000/#download [File for L1000 information: LINCS_Gene_Experssion_signatures_CD.csv.gz and file for 166-bit MACCS chemical structure fingerprint matrices: MACCS_bitmatrix.csv.gz] with processing information detailed by Wang *et al*.^12^. The rat BUN level datasets analysed during this study are available from the DrugMatrix repository at https://ntp.niehs.nih.gov/results/drugmatrix/index.html. The hyperparameters for our best GP model and closest competitor model generated with LightGBM are detailed in Supplementary Table S4.
