# Supplementary material for: Using human in vitro transcriptome analysis to build trustworthy machine learning models for prediction of animal drug toxicity
Source: Sci Rep. 2020 Jun 12;10:9522. doi: 10.1038/s41598-020-66481-0 (PMC7293302; doi:10.1038/s41598-020-66481-0)
Supplement: Supplementary file 1 — Supplemental information. [file 41598_2020_66481_MOESM1_ESM.docx]

## **Supplementary Information**

Title: Using human *in vitro* transcriptome analysis to build trustworthy machine learning models for prediction of animal drug toxicity

Authors: Laura-Jayne Gardiner^1*^, Anna Paola Carrieri^1^, Jenny Wilshaw^1^, Stephen Checkley^2^, Edward O. Pyzer-Knapp^1^, Ritesh Krishna^1*^

Affiliations: *^1^IBM Research UK, Sci-Tech Daresbury, Warrington, UK*

*^2^STFC Daresbury Lab., Warrington, UK*

Laura-Jayne Gardiner: Laura-Jayne.Gardiner@ibm.com

Anna Paola Carrieri: acarrieri@uk.ibm.com

Jenny Wilshaw^1^: jwilshaw@rvc.ac.uk

Stephen Checkley^2^: scheckley@gmail.com

Edward O. Pyzer-Knapp^1^: epyzerk3@uk.ibm.com

Ritesh Krishna: Ritesh.krishna@uk.ibm.com

^*^Authors for correspondence:

Laura-Jayne Gardiner: Laura-Jayne.Gardiner@ibm.com

IBM Research UK,

Sci-Tech Daresbury,

Warrington,

UK

Ritesh Krishna: Ritesh.krishna@uk.ibm.com

IBM Research UK,

Sci-Tech Daresbury,

Warrington,

UK

**Figure S1. Block diagram to show analysis pipeline for dataset preparation for ML prediction of BUN levels in rats from L1000 gene expression and chemical structure information.**

**Table S1. ML analyses to predict BUN levels in rats: comparing commonly used deterministic methods (without tSVDh) to our best GP model.** Showing the results from the analysis combining chemical structure and L1000 gene expression data as feature sets and comparing the commonly used methods to our best GP model (produced using tSVDh). *Mean ± SD training cross validation score (MAE) **Weighted RMSE

| Regressor | Linear REGRESSION | RANDOM FOREST | SVM | KNN | XGBoost | Gradient boosting | light  gbm | Gaussian Process + tsvd hierarchical |
| --- | --- | --- | --- | --- | --- | --- | --- | --- |
| **Training set MAE score using best parameters** | 1.000  *3.997  ±0.506 | 1.488  *2.882  ±1.179 | 0.394  *2.776  ±1.537 | 0.000  *3.730  ±1.771 | 2.012  *2.878  ±1.731 | 1.832  *2.577  ±1.552 | 0.501  *3.334 ±1.060 | **0.000** |
| **Test set MAE score using best parameters** | 3.676 | 1.900 | 1.997 | 2.496 | 2.200 | 2.151 | 1.544 | **1.710** |
| **Test set RMSE score using best parameters** | 4.629 | 3.519 | 3.479 | 3.683 | 3.406 | 3.654 | 2.146 | **2.419**  ****1.528** |
| **Test set r2 score using best parameters** | -0.600 | 0.075 | 0.096 | -0.013 | 0.134 | 0.003 | 0.656 | **0.661** |

**Table S2. ML analyses to predict BUN levels in rats: comparing commonly used deterministic methods (with tSVDh) to our best GP model.** Showing the results from the analysis combining chemical structure and L1000 gene expression data as feature sets and comparing the commonly used methods to our best GP model (produced using tSVDh). *Mean ± SD training cross validation score (MAE) **Weighted RMSE

| Method | tsvd hierarchical | | | | | | | tsvd hierarchical |
| --- | --- | --- | --- | --- | --- | --- | --- | --- |
| **Regressor** | LINEAR REGRESSION | RANDOM FOREST | SVM | KNN | XGBOOST | GRADIENT BOOSTING | LIGHT  GBM | **GAUSSIAN PROCESS** |
| **Training set MAE score using best parameters** | 1.791  *3.419  ±0.785 | 1.318  *3.017  ±1.421 | 0.334  *2.808  ±1.571 | 0.000  *3.364  ±1.510 | 1.096  *3.316  ±1.161 | 1.993  *2.957  ±1.722 | 1.415  *3.065 ±1.354 | **0.000** |
| **Test set MAE score using best parameters** | 2.343 | 2.055 | 1.877 | 2.029 | 2.138 | 2.209 | 2.181 | **1.710** |
| **Test set RMSE score using best parameters** | 3.603 | 3.426 | 3.327 | 3.292 | 3.171 | 3.679 | 3.460 | **2.419**  ****1.528** |
| **Test set r2 score using best parameters** | 0.041 | 0.133 | 0.182 | 0.199 | 0.257 | 0.000 | 0.115 | **0.661** |

**Table S3. Parameter training during hyperparameter optimization for comparison of commonly used deterministic ML methods to our best GP model.** Showing the parameters that were tuned using Grid Search and the range of trialled hyperparameters.

| Regressor | hyperparameter tuning |
| --- | --- |
| LINEAR REGRESSION | fit_intercept:[True,False]  normalize:[True,False]  copy_X:[True, False] |
| RANDOM FOREST | criterion: ['mse', 'mae']  min_samples_leaf: [1, 2, 3, 4, 5, 6, 7, 8, 9, 10]  max_depth: [1, 2, 3, 4, 5, 6, 7, 8, 9, 10]  min_samples_split: [2, 5, 10] |
| SVM | kernel: ['linear', 'poly', 'rbf']  max_iter: [int(x) for x in np.linspace(start=100, stop=4000, num=10)]  gamma: scipy.stats.expon(scale=.1)  C: [1, 2, 3, 4, 5, 6, 7, 8, 9, 10]  degree: [0, 1, 2, 3, 4, 5, 6]  coef0: scipy.stats.expon(scale=1) |
| KNN | n_neighbors: [1, 2, 3, 4, 5, 6, 7, 8, 9, 10, 11, 12, 13, 14, 15, 16, 17, 18, 19, 20]  leaf_size: [1, 2, 3, 4, 5]  weights:['uniform', 'distance']  algorithm:['auto', 'ball_tree','kd_tree','brute'] |
| XGBOOST | max_depth: [2, 3, 4, 5, 6, 7, 8, 9, 10, 15, 20, 25]  subsample: [0.2, 0.5, 0.6, 0.7, 0.8, 0.9, 1]  learning_rate: scipy.stats.expon(scale=1)  min_child_weight: scipy.stats.expon(scale=10)  max_delta_step: [0, 1, 2]  early_stopping_rounds: [10]  objective: ['reg:linear']  gamma: scipy.stats.expon(scale=20)  n_estimators: [50, 150, 200, 250, 300, 350, 400, 450, 500] |
| GRADIENT BOOSTING | criterion: ['mse', 'mae', 'friedman_mse']  min_samples_leaf: [50, 100, 200]  max_depth: [3, 5, 6, 7, 8, 10]  min_samples_split: [100, 200, 300, 400, 500]  max_features: ['sqrt']  learning_rate: [0.05, 0.1, 0.2]  n_estimators: [20, 40, 60, 80]  subsample: [0.2, 0.5, 0.6, 0.7, 0.8, 0.9, 1] |
| LIGHT  GBM | num_leaves: [10, 20, 50, 100, 200]  subsample: [0.2, 0.5, 0.6, 0.7, 0.8, 0.9, 1]  min_data_in_leaf: [10, 25, 50, 75, 100]  max_depth: [3, 5, 6, 7, 8, 10, 15, 20, 25]  learning_rate: scipy.stats.expon(scale=1)  n_estimators: [50, 150, 200, 250, 300, 350, 400, 450, 500] |
| GAUSSIAN PROCESS | normalize_y:[True,False]  copy_X_train:[True, False]  alpha: [1e-2, 1e-4, 1e-6, 1e-8, 1e-10, 1e-12]  n_restarts_optimizer: [10]  WhiteKernel(noise_level=1e-7)  length_scale: np.ones(X_train.shape[1]) |

**Table S4. Best performing regression models from ML analyses to predict BUN levels.** Detailing the parameter sets used for our best performing GP model plus its closest competitor model generated using LighGBM. Both models were trained using L1000 gene expression data plus chemical structure information. The GP model was trained after use of tSVDh while LightGBM was more predictive using the full feature set.

| Target phenotype | REGRESSOR | hyperparameters |
| --- | --- | --- |
| **MODEL 1**  **BUN level** | Gaussian Process | kernel=’RBF’, alpha=1e-10, optimizer=’fmin_l_bfgs_b’, n_restarts_optimizer= 0, normalize_y= False, copy_X_train= True, random_state=42,  WhiteKernel(noise_level=1e-7)  length_scale: np.ones(X_train.shape[1]) |
| **MODEL 2**  **BUN level** | LightGBM | random_state=42, boosting_type='gbdt', class_weight=None, colsample_bytree=1.0, importance_type='split', learning_rate=0.3570361159233177, max_depth=25, min_child_samples=20, min_child_weight=0.001, reg_lambda=0.0, silent=True, subsample=1, subsample_for_bin=200000, subsample_freq=0, num_leaves=20, n_estimators=350, min_data_in_leaf=50 |
